# Adsorption Studies of the Gram-Negative Bacteria onto Nanostructured Silicon Carbide

**DOI:** 10.1007/s12010-014-1374-4

**Published:** 2014-11-20

**Authors:** Andrzej Borkowski, Mateusz Szala, Tomasz Cłapa

**Affiliations:** 1 Faculty of Geology, University of Warsaw, Żwirki i Wigury 93, 02-089 Warsaw, Poland; 2 Faculty of Advanced Technologies and Chemistry, Military University of Technology, Kaliskiego 2, 00-908 Warsaw, Poland; 3 Department of General and Environmental Microbiology, Poznań University of Life Sciences, Szydłowska 50, 60-656 Poznań, Poland

**Keywords:** Adsorption, Bacteria, Nanofibers SiC, Nanorods SiC, *Pseudomonas putida*, Silicon carbide

## Abstract

In this study, we demonstrated a significant adsorption of *Pseudomonas putida* bacteria onto aggregates of nanofibers (NFSiC) and nanorods (NRSiC) of silicon carbide (SiC) in aqueous suspensions. Langmuir and Freundlich isotherms were used to quantify adsorption affinities. It was found that adsorption of the bacteria strongly depended on the structure of the silicon carbide and the pH of the aqueous solution, which affected the isoelectric point of both the silicon carbide and the bacterial cells. The strongest affinity of bacteria was noted in the case of NRSiC aggregates. Affinity was inversely proportional to pH. Similarly, the adsorption of bacteria to the surface of the aggregates increased with decreasing pH. For NFSiC, the affinity of the bacteria for the surface of the aggregates was also inversely proportional to pH. However, adsorption increased at higher pH values. This discrepancy was explained by microscopic analysis, which showed that the bacterial cells were both adsorbed onto and trapped by NFSiC. The adsorption of bacteria onto a micrometric silicon carbide reference material was significantly smaller than adsorption onto nanostructured SiC.

**Electronic supplementary material:**

The online version of this article (doi:10.1007/s12010-014-1374-4) contains supplementary material, which is available to authorized users.

## Introduction

In view of the growing interest in nanostructured materials based on carbon or carbon-containing compounds, the study of the interaction between such materials and living cells is a worthwhile pursuit. This interest stems from both the potential cytotoxic properties of such materials, as well as their potential applications for water purification technologies [1–3]. Also of interest is the possibility of modifying the surface of various materials to exhibit antibacterial properties. Finally, there is also a significant potential risk of the presence of nanostructured materials in the environment [1].

A majority of the studies of interactions between bacteria and nanostructured materials concern single-walled and multiwalled carbon nanotubes, graphene, fullerenes [4–7], and modified carbon materials containing metals such as zinc [8]. Some of these studies also analyzed the adsorption of bacteria onto the surface of such materials. This is an important aspect of research that relates to the potential applications of these materials in water purification methods [9–11]. A similar research is focused not only on nanostructured materials but also on biogeochemical interactions between minerals and bacteria, including biofouling, biocorrosion, and mechanisms of biofilm formation [12, 13]. In this context, studies have shown the possibility of adsorption of Gram-positive and Gram-negative bacteria to minerals such as quartz, corundum, and iron-containing minerals. The adsorption of bacteria to clay minerals also was studied [14], which was not easy due to the difficulty of separating unadsorbed bacteria and mineral particles. Similar difficulties may arise when examining the adsorption of bacteria onto aggregates of nanostructures in aqueous suspensions, but the appropriate use of reagents that increase the density of the aqueous environment allows the separation and measurement of unadsorbed bacteria [14].

The literature describing the adsorption and survival of bacteria on the surface of nanostructured materials is largely focused on modified carbon materials and nanostructured carbon materials, while there are no similar studies on nanostructured silicon carbide (SiC). Silicon carbide appears to be completely inert with respect to biological systems; however, like other materials, it can interact with living cells when nanostructured. The literature describes two basic effects of these interactions: mechanical damage and oxidative stress caused by the presence of highly reactive chemical species on the surface of the nanostructures [15–17].

Because new routes of combustion synthesis of SiC nanofibers (NFSiC) and SiC nanorods (NRSiC) were developed, the study of cytotoxicity and the interactions between these materials and living organisms is of great interest. Research conducted by Szala and Borkowski [18] showed a significant toxicity of NFSiC and NRSiC against the bacterium *Pseudomonas putida*, which was manifested by mechanical damage of cells, reduction of dehydrogenase activity, and a decrease in biochemical activity as measured by CO_2_ production. The aim of the present study was to investigate the adsorption of the Gram-negative bacterium *P. putida* onto the surface of aggregates of SiC nanofibers and nanorods in aqueous suspensions. The adsorption tests were also performed using a micrometric silicon carbide (μmSiC) reference material to verify the hypothesis that the adsorption of bacteria depends on the morphology and texture of the SiC.

## Materials and Methods

### Nanomaterials and μmSiC

NFSiC was prepared by combustion synthesis [19]. The combusted mixture was prepared by dry mixing powders of calcium silicide (CaSi_2_) and poly(tetrafluoroethene) (PTFE) in a ceramic mortar. After cold pressing into a cylindrical pellet, 5 g of the sample was placed in a graphite crucible and put into a stainless steel autoclave (400 cm^3^ in volume), which was subsequently filled with argon at an initial pressure of 1.0 MPa. The combustion process was initiated with an electrically heated resistance wire. The solid combustion products were removed from the autoclave with water. The suspension was filtered and the deposit obtained was purified in a three-step process: heating in 95 % H_2_SO_4_, calcination in air (700 °C), and, finally, heating in 30 % NaOH and washing with water. The NFSiC prepared using this method was used in all experiments. NRSiC was also prepared by the combustion route, but the starting mixture comprised fluorinated graphite (CF) and aluminum-silicon (AlSi) [20]. Purification was carried out following the same procedure, but the step of heating in H_2_SO_4_ was omitted. X-ray powder diffraction (XRD) spectra of NFSiC and NRSiC were measured (D5000 diffractometer—Siemens, Cu Kα radiation) in the range of 2θ from 10° to 95°. Silicon carbide micrometric powder (μmSiC) was purchased from Sigma-Aldrich and used without purification.

**Fig. 1:**
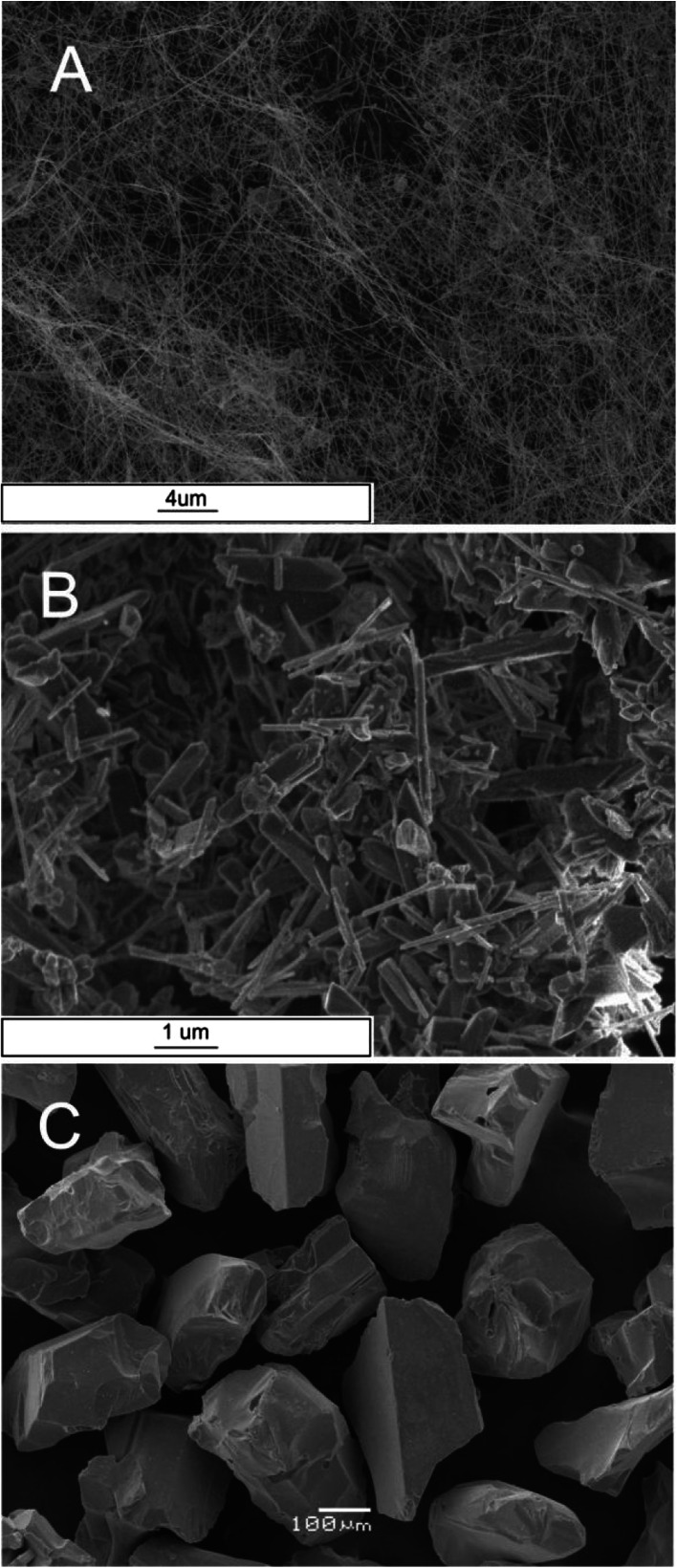
SEM images of silicon carbide. **a** SiC nanofibers, **b** SiC nanorods, and **c** micrometric SiC

The BET specific surface area of the materials used in the studies also was estimated. This measurement was performed using the standard apparatus for measuring surface area and porosity, an ASAP 2020 (Micromeritics). The determination was carried out at liquid nitrogen temperature (−195.8 °C). Nitrogen with a purity of 6.0 N was used as an adsorbate. The point of zero charge (PZC) of the investigated materials was analyzed by potentiometric mass titrations [21]. The example of obtained mass titration curves is presented in supplementary material. The PZC of bacteria was analyzed according to [22]. This procedure is similar to potentiometric mass titrations and has been tested in both bacterial cells and isolated cell walls [22]. Scanning electron microscopy (SEM) images of the investigated materials are presented in Fig. 1. Properties of the investigated materials are shown in Table 1.

**Table 1:** Selected properties of investigated materials

Material/bacteria	Dimensions length × width	BET [m^2^ g^−1^]	PZC
μmSiC	500 × 250 μm	20	2.7
NFSiC	>1000 × 5–50 nm	125	2.8
NRSiC	100–2400 × 50–300 nm	130	3.5
*P. putida*	–	–	2.8

*BET* specific surface area measured by BET isotherms of adsorption of nitrogen, *PZC* point of zero charge

### Microorganisms and Media

The strain of *P. putida* was obtained from our own collection of microorganisms isolated from samples of organic soils (Geomicrobiology Laboratory, Faculty of Geology, University of Warsaw, Poland). Taxonomic affiliation was confirmed by DNA sequence analysis of the 16S ribosomal RNA gene. The bacteria were cultivated in both liquid and solid nutrient media (pH 7.5) comprising the following (g L^−1^): glucose, 10; peptone, 5; yeast extract, 2; NaCl, 4; and agar (in the case of solid medium), 20. The media were sterilized by autoclaving at 121 °C for 15 min.

### Protein Measurements

**Fig. 2:**
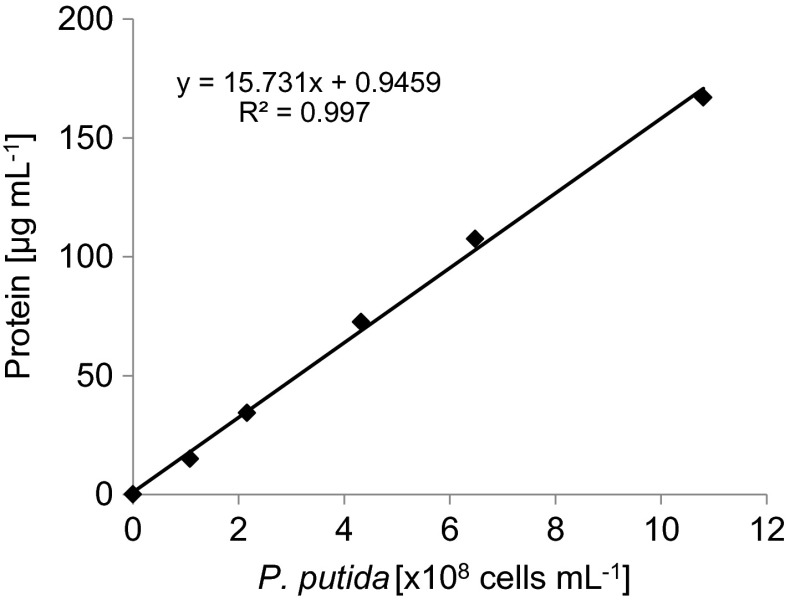
Correlation between protein concentration and number of bacterial cells

To analyze the number of adsorbed bacteria without using the cultivation method (in respect of the cytotoxic properties of nanostructured SiC), the correlation between protein content and bacterial counts was plotted. The measurement was performed as follows. Five different suspensions of bacteria (1–10 × 10^8^ cells mL^−1^) were put (1 mL) into glass tubes and 5 mL of reagent (49 mL 2 % Na_2_CO_3_ in 0.1 M NaOH + 0.5 mL 2 % potassium sodium tartrate + 0.5 mL 1 % CuSO_4_) was added. After 5 min, 0.2 mL of Folin-Ciocalteu reagent (POCH, Gliwice, Poland) was added and immediately mixed. After 5 min, 0.2 mL 6 M NaOH was added and mixed, then the absorbance of the colored solution was measured spectrophotometrically at 670 nm (Genesys 10Vis, Thermo). The correlation is presented in Fig. 2.

### Adsorption Tests

Adsorption tests were conducted in phosphate buffer (1/15 M) at three pH values: 3.0, 6.8, and 9.0. pH was adjusted with small aliquots of NaOH (ca. 20 μL, 6 M) or H_3_PO_4_ (ca. 20 μL, 80 %). Adsorption was measured as follows. Twenty milligrams of NFSiC or NRSiC, or 50 mg of μmSiC, was mixed with 2 mL of buffer containing 0–10 × 10^8^ *P. putida* cells mL^−1^. Next, the suspensions were shaken (120 rpm) for 3 h at 25 °C. After mixing, 1 mL of the suspension was placed into an Eppendorff tube and 0.3 mL of sucrose (60 %) was added. Then, the mixture was centrifuged for 2 min at 4000 rpm to remove the SiC. One milliliter of supernatant containing unadsorbed bacteria was used to determine the protein concentration as described above. The number of adsorbed bacteria was calculated from the difference between the number of bacteria in suspension before and after adsorption. The adsorption tests were conducted in triplicate. The parameters of Langmuir and Freundlich isotherms were calculated to quantify the affinity of bacteria for the SiC materials at three different pH values. The Langmuir isotherm is described by the following equation:

1 $$ n=\frac{A_{\mathrm{m}}\times {K}_{\mathrm{L}}\times {C}_{\mathrm{eq}}}{1+{K}_{\mathrm{L}}\times {C}_{\mathrm{eq}}} $$

where *n* is the amount of adsorbed bacteria (×10^10^ cells g^−1^), *A*_m_ is the maximal number of adsorbed bacteria (×10^10^ cells g^−1^), *K*_L_ is the Langmuir constant, and *C*_eq_ is the equilibrium bacterial concentration (×10^8^ cells g^−1^).

The Freundlich isotherm is described by the equation:

2 $$ n={K}_{\mathrm{F}}\times {C_{\mathrm{eq}}}^b $$

where *K*_F_ is the constant of the isotherm, *b* is a parameter that may have a value in the range <0;1>, and *n* and *C*_eq_ are as described for the Langmuir isotherm.

To fit the experimental data to the Langmuir model, a statistical spreadsheet (STATISTICA) was applied using the method of least squares for nonlinear models, while the Freundlich model was fitted to the experimental data using a linearized isotherm:

3 $$ \log n=b\times \log {C}_{\mathrm{eq}}+ \log {K}_{\mathrm{F}}. $$

### Microscopy

Samples of NFSiC and NRSiC with adsorbed bacteria were placed on an aluminum plate and coated with gold. Pure SiC nanostructures were observed without coating. SEM images were obtained with a JEOL JSM-6380LA microscope.

Fluorescence microscopy images of bacteria attached to aggregates of NFSiC and NRSiC were obtained after drying samples of nanoSiC with bacteria on a clean glass plate and staining with acridine orange. The glass plate was then rinsed with water and, after drying, was viewed under epifluorescence microscopy with a B-filter.

## Results and Discussion

SEM images (Fig. 1) reveal that investigated substances have different morphologies. Figure 1a showed nanofibers with 10–100 nm in diameter and length exceed 10 μm. Nanofibers are relatively pure and did not contain three-dimensional crystals or nanorods. Nanorods visible on Fig. 1b are homogenic in shape and 100–500 nm in diameter and length about 1–2 μm. The nanorod surface is relatively smooth. Figure 1c showed that the sample have no nanostructural morphology. The sample is built from irregular polygonal particles with an average diameter about 500 μm.

During preliminary tests, the relationship between the number of bacteria in the suspension and the concentration of protein was plotted (Fig. 2). The data indicate a significant correlation between the two variables (*R* ^*2*^ = 0.997, *p* < 0.001). Thus, the number of bacteria in the suspension can be estimated accurately by the protein concentration, which greatly facilitates the measurement of bacterial adsorption onto the surface of nanostructured materials. In some studies, determinations of bacterial counts after adsorption were obtained by the cultivation method [8]. However, this approach is not suitable in cases where the material exhibits cytotoxic properties. Earlier studies [18] indicated a possible significant toxicity of nanostructured SiC to *P. putida*; hence, our methodology is justified.

### Adsorption of Bacteria

**Fig. 3:**
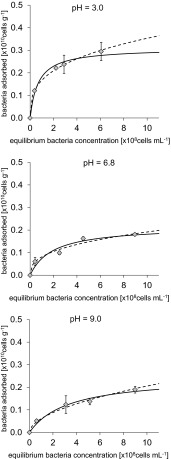
Isotherms of adsorption of *P. putida* on μmSiC in three different values of pH. *Symbols* represent experimental data, *solid curve line*—Langmuir model, and *dashed curve line*—Freundlich model. Standard deviation has been marked

**Fig. 4:**
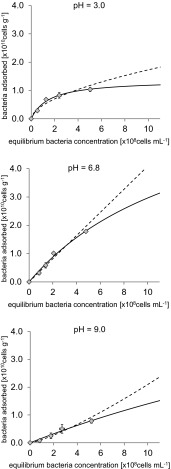
Isotherms of adsorption of *P. putida* on NFSiC in three different values of pH. *Symbols* represent experimental data, *solid curve line*—Langmuir model, and *dashed curve line*—Freundlich model. Standard deviation has been marked

**Fig. 5:**
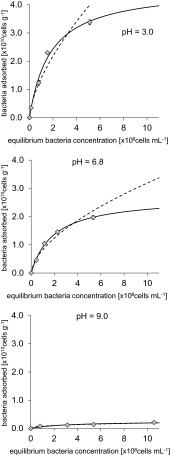
Isotherms of adsorption of *P. putida* on NRSiC in three different values of pH. *Symbols* represent experimental data, *solid curve line*—Langmuir model, and *dashed curve line*—Freundlich model. Standard deviation has been marked

The isotherms of bacterial adsorption onto the surface of μmSiC and aggregates of NRSiC and NFSiC are shown in Figs. 3, 4, and 5, respectively. The relationship between the equilibrium number of bacteria in the suspension and the number of adsorbed cells was very well described by the Langmuir and Freundlich adsorption isotherm equations. The Langmuir model in studies on the adsorption of bacterial cells to natural materials, such as clay minerals and iron oxides, was applied by Jiang et al. [14]. The use of these isotherms in the present study gave high values of correlation coefficients (*r*), as shown in Table 2. The results showed that the adsorption of bacteria onto the surface of μmSiC was significantly lower than that in the case of nanostructured SiC. Additionally, the strong dependence between the adsorption of bacteria and pH was observed, particularly in the case of adsorption onto NRSiC. Quantitative comparison of bacterial adsorption onto the SiC materials was possible due to the computed Langmuir isotherm parameters (Table 2). The obtained values of *A*_m_ (maximal number of bacteria that may be adsorbed) for the adsorption of bacteria on the surface of μmSiC at pH 3.0, 6.8, and 9.0 reached 0.31, 0.22, and 0.25 × 10^10^ cells g^−1^, respectively, while the values of *A*_m_ for adsorption onto the surface of NFSiC and NRSiC aggregates were an order of magnitude greater, except for the case of adsorption of bacteria onto NRSiC at pH 9.0, in which case the *A*_m_ reached 0.25 × 10^10^ cells g^−1^. The largest *A*_m_ value (8.26 at 10^10^ × g^−1^) was determined in the case of bacterial adsorption onto NFSiC at pH 9.0. However, this result may be due to an artifact because the adsorption isotherms for NFSiC at pH 6.8 and 9.0 were linear or nearly linear and the Freundlich isotherms had no physical sense (Fig. 4). For example, the value of parameter *b* for the Freundlich adsorption isotherm at pH 9.0 was determined to be 1.26, which indicates an increasing trend of isotherm. This problem could not be solved by increasing of concentration of bacteria in experiments due to the possibility of bacterial flocculation. In the case of adsorption of bacteria onto the surface of NRSiC aggregates (Fig. 5), at pH = 3.0, the value of *A*_m_ reached 4.61 × 10^10^ cells g^−1^, while at pH = 9.0, the value was 0.25 × 10^10^ cells g^−1^.

**Table 2:** Parameters of Langmuir and Freundlich isotherms of adsorption of bacteria on investigated materials and correlation coefficients (*r*)

Material	pH	Langmuir model			Freundlich model		
		*A*_m_ [×10^10^ cells g^−1^]	*K*_L_	*r*	*K*_F_	*b*	*r*
μmSiC	3.0	0.31	1.44	0.99	0.17	0.32	0.99
	6.8	0.22	0.52	0.97	0.08	0.37	0.95
	9.0	0.25	0.30	0.99	0.06	0.53	0.98
NFSiC	3.0	1.37	0.65	0.99	0.48	0.56	0.90
	6.8	7.32	0.07	0.99	0.41	0.99	0.97
	9.0	8.26	0.02	0.99	0.11	1.26	0.97
NRSiC	3.0	4.61	0.56	0.99	1.39	0.64	0.97
	6.8	2.69	0.52	0.99	0.82	0.59	0.95
	9.0	0.25	0.31	0.97	0.08	0.38	0.97

### Effect of pH on the Affinity Between Bacteria and SiC Materials

**Fig. 6:**
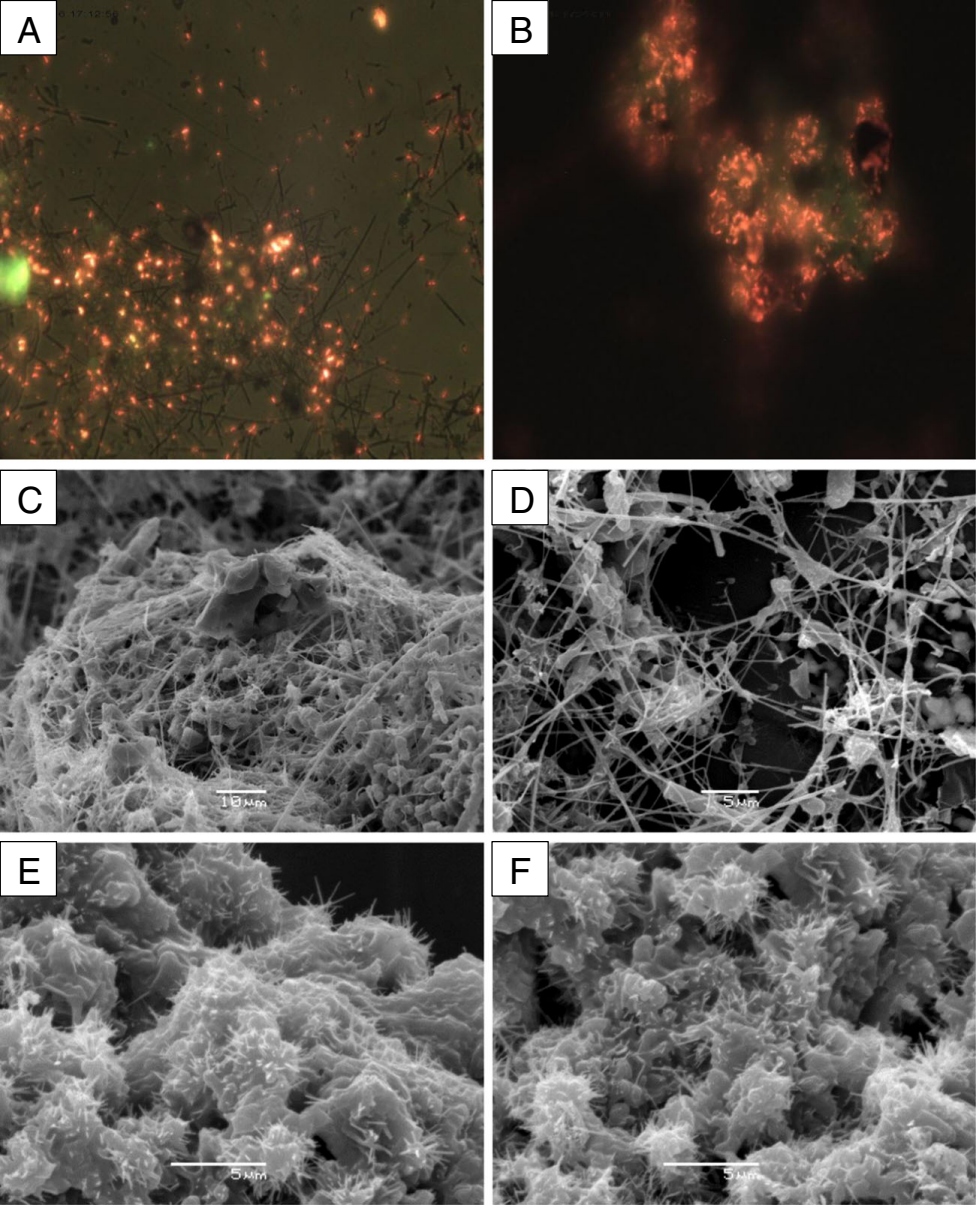
Bacteria adsorbed on nanostructured SiC. **a** and **b** bacteria adsorbed on aggregates of NFSiC and NRSiC, respectively. Fluorescence microscopy (×600), orange acridine staining. **c**–**f** SEM images of bacteria attached to NFSiC (**c**, **d**) and NRSiC (**e**, **f**)

For NFSiC and NRSiC, a dependence of bacterial adsorption on pH was observed. This phenomenon can be analyzed based on the values of the parameter *K*_L_ of the Langmuir model, which are shown in Table 2. These values can be interpreted as the degree of affinity between the bacteria and the tested materials [14]. Therefore, a higher *K*_L_ value indicates a higher affinity of bacteria for the tested material under the given conditions. The *K*_L_ values for the adsorption of bacteria indicated the inversely proportional dependence of affinity on pH. This indicates, therefore, a decrease in the affinity of bacteria for the investigated material with increasing pH. The highest values of *K*_L_ were determined at the lowest pH: 1.44, 0.65, and 0.56 for μmSiC, NFSiC, and NRSiC, respectively. The interpretation of these data should be partially assessed in light of the PZC values of the SiC materials and *P. putida* (Table 1). Taking into account the PZC values of NRSiC and bacteria, it can be seen that the lower pH causes a stronger interaction between the bacteria and the material. Below the PZC, the surface charge of the bacterial cells and NRSiC is positive. At pH = 3, the NRSiC surface charge was positive, while the surface charge of the bacteria was negative. For this case, the parameters of the Langmuir isotherm showed that the bacteria had a high affinity for the NRSiC aggregates. However, in the case of NFSiC, a reduction in the affinity of the bacteria for the tested material (based on *K*_L_) and an increase in the number of adsorbed bacteria (*A*_m_) with increasing pH values were found. It is possible, however, that in the case of fibrous NFSiC structures, bacteria may simply be entrapped in the fibers, thus resulting in anomalously high *A*_m_ values. The efficiency of this process may increase with increasing pH because at higher pH values, these bacteria were more widely dispersed in the aqueous suspension. Images obtained by fluorescence microscopy and SEM confirm the hypothesis about the “trapping” of bacteria (Fig. 6). In the case of the NRSiC, it can be observed that the bacteria strictly adhered to aggregates of the material, as can be clearly seen in fluorescence microscopy images. Similarly, SEM images showed cells forming a compact structure on the surface of NRSiC aggregates. In the case of NFSiC, it can be seen that, in contrast, cells were entrapped in the nanofiber structure, which is particularly visible in the SEM images.

Studies describing the adsorption of bacteria to minerals characterized by different values of PZC show that the mechanism of adsorption is controlled by the surface charge of the material and cells. In the study by Ams et al. [23], adsorption of Gram-positive *Bacillus subtilis* and Gram-negative *Pseudomonas mendocina* onto the surface of Fe oxyhydroxide-coated and uncoated quartz grains as a function of pH was studied. Adsorption appeared to be controlled by the surface charge of the bacteria and mineral surface. Bacterial adsorption onto the surface of nanostructures was shown by Kang et al. [4] and Singh et al. [24]. Similarly, the ability to create a biofilm and adsorption of the bacterial cells to the surface of mineral materials, including carbon materials and modified clay minerals, were also demonstrated [8, 10, 12–14]. These studies also indicated the important role of pH and ionic strength of the solution as significant factors affecting the adsorption of bacterial cells onto the surface of minerals.

## Conclusion

In this study, it has been shown that the adsorption of *P. putida* onto the surface of SiC strongly depends on the morphology of SiC and pH. NFSiC not only allows adsorption of the bacterial cells but also “traps” these cells, which may be particularly important in the case of SiC nanofibers. The results showed a strong dependence of the binding of bacteria on pH, which is indicative of the fact that adsorption was partially controlled by both the surface charge of the SiC and the bacterial cells. Such conclusions can be drawn on the basis of the determined isotherm parameters, primarily on that of the *K*_L_ parameter, which reflects the affinity between cells and the test material. Adsorption of bacteria onto the surface of nanostructured SiC in water suspensions can be of great practical importance in view of the development of highly durable and chemically inert nanomaterials in water treatment technologies or filters.

## Electronic Supplementary Material

Below is the link to the electronic supplementary material.

ESM 1 (DOCX 60 kb)
