# Supplementary material for: Adsorption Studies of the Gram-Negative Bacteria onto Nanostructured Silicon Carbide
Source: Appl Biochem Biotechnol. 2014 Nov 20;175(3):1448–59. doi: 10.1007/s12010-014-1374-4 (PMC4318990; doi:10.1007/s12010-014-1374-4)
Supplement: Supplementary file 1 — (DOCX 60 kb) [file 12010_2014_1374_MOESM1_ESM.docx]

Fragment of mass titration curves. The measurement was realized according to Bourikas et al. [21]. The titration curves for µmSiC was very similar to NFSiC.
